# Optimal blood tau species for the detection of Alzheimer’s disease neuropathology: an immunoprecipitation mass spectrometry and autopsy study

**DOI:** 10.1007/s00401-023-02660-3

**Published:** 2023-12-30

**Authors:** Laia Montoliu-Gaya, Michael L. Alosco, Eukyung Yhang, Yorghos Tripodis, Daniel Sconzo, Madeline Ally, Lana Grötschel, Nicholas J. Ashton, Juan Lantero-Rodriguez, Mathias Sauer, Bárbara Gomes, Johanna Nilsson, Gunnar Brinkmalm, Michael A. Sugarman, Hugo J. Aparicio, Brett Martin, Joseph N. Palmisano, Eric G. Steinberg, Irene Simkin, Katherine W. Turk, Andrew E. Budson, Rhoda Au, Lindsay Farrer, Gyungah R. Jun, Neil W. Kowall, Robert A. Stern, Lee E. Goldstein, Wei Qiao Qiu, Jesse Mez, Bertrand Russell Huber, Victor E. Alvarez, Ann C. McKee, Henrik Zetterberg, Johan Gobom, Thor D. Stein, Kaj Blennow

**Affiliations:** 1 Department of Psychiatry and Neurochemistry, Institute of Neuroscience and Physiology, The Sahlgrenska Academy at the University of Gothenburg, Mölndal, Sweden; 2 Boston University Alzheimer’s Disease Research Center and CTE Center, Boston University, Chobanian and Avedisian School of Medicine, Boston, MA 02118 USA; 3 Department of Neurology, Boston University, Chobanian and Avedisian School of Medicine, Boston, MA 02118 USA; 4 Department of Biostatistics, Boston University School of Public Health, Boston, MA 02118 USA; 5 University of Arizona, Tucson, AZ USA; 6 Centre for Age-Related Medicine, Stavanger University Hospital, Stavanger, Norway; 7 Department of Old Age Psychiatry, Maurice Wohl Clinical Neuroscience Institute, King’s College London, London, UK; 8 NIHR Biomedical Research Centre for Mental Health and Biomedical Research Unit for Dementia at South London & Maudsley NHS Foundation, London, UK; 9 Department of Neurology, Medical University of South Carolina, Charleston, SC 29425 USA; 10 Biostatistics and Epidemiology Data Analytics Center, Boston University School of Public Health, Boston, MA 02118 USA; 11 Department of Medicine, Boston University, Chobanian and Avedisian School of Medicine, Boston, MA 02118 USA; 12 VA Boston Healthcare System, U.S. Department of Veteran Affairs, Jamaica Plain, MA 02130 USA; 13 Department of Epidemiology, Boston University School of Public Health, Boston, MA 02118 USA; 14 Department of Anatomy and Neurobiology, Boston University School of Medicine, Boston, MA 02118 USA; 15 Department of Neurosurgery, Boston University School of Medicine, Boston, MA 02118 USA; 16 Department of Psychiatry and Ophthalmology, Boston University School of Medicine, Boston, MA 02118 USA; 17 Department of Biomedical, Electrical and Computer Engineering, Boston University College of Engineering, Boston, MA 02215 USA; 18 Department of Pharmacology and Experimental Therapeutics, Boston University, Chobanian an Avedisian School of Medicine, Boston, MA 02118 USA; 19 Department of Psychiatry, Boston University, Chobanian and Avedisian School of Medicine, Boston, MA 02118 USA; 20 VA Bedford Healthcare System, U.S. Department of Veteran Affairs, Bedford, MA 01730 USA; 21 Department of Pathology and Laboratory Medicine, Boston University School of Medicine, Boston, MA 02118 USA; 22 Clinical Neurochemistry Laboratory, Sahlgrenska University Hospital, Mölndal, Sweden; 23 Department of Neurodegenerative Disease, Queen Square Institute of Neurology, University College London, London, UK; 24 UK Dementia Research Institute, University College London, London, UK; 25 Hong Kong Center for Neurodegenerative Diseases, Hong Kong, China; 26 UW Department of Medicine, School of Medicine and Public Health, Madison, WI USA

**Keywords:** Blood, Phosphorylated tau, Alzheimer’s disease, Autopsy, Biomarkers, Mass spectrometry

## Abstract

Plasma-to-autopsy studies are essential for validation of blood biomarkers and understanding their relation to Alzheimer’s disease (AD) pathology. Few such studies have been done on phosphorylated tau (p-tau) and those that exist have made limited or no comparison of the different p-tau variants. This study is the first to use immunoprecipitation mass spectrometry (IP-MS) to compare the accuracy of eight different plasma tau species in predicting autopsy-confirmed AD. The sample included 123 participants (AD = 69, non-AD = 54) from the Boston University Alzheimer’s disease Research Center who had an available ante-mortem plasma sample and donated their brain. Plasma samples proximate to death were analyzed by targeted IP-MS for six different tryptic phosphorylated (p-tau-181, 199, 202, 205, 217, 231), and two non-phosphorylated tau (195–205, 212–221) peptides. NIA-Reagan Institute criteria were used for the neuropathological diagnosis of AD. Binary logistic regressions tested the association between each plasma peptide and autopsy-confirmed AD status. Area under the receiver operating curve (AUC) statistics were generated using predicted probabilities from the logistic regression models. Odds Ratio (OR) was used to study associations between the different plasma tau species and CERAD and Braak classifications. All tau species were increased in AD compared to non-AD, but p-tau217, p-tau205 and p-tau231 showed the highest fold-changes. Plasma p-tau217 (AUC = 89.8), p-tau231 (AUC = 83.4), and p-tau205 (AUC = 81.3) all had excellent accuracy in discriminating AD from non-AD brain donors, even among those with CDR < 1). Furthermore, p-tau217, p-tau205 and p-tau231 showed the highest ORs with both CERAD (OR_p-tau217_ = 15.29, OR_p-tau205_ = 5.05 and OR_p-tau231_ = 3.86) and Braak staging (OR_p-tau217_ = 14.29, OR_p-tau205_ = 5.27 and OR_p-tau231_ = 4.02) but presented increased levels at different amyloid and tau stages determined by neuropathological examination. Our findings support plasma p-tau217 as the most promising p-tau species for detecting AD brain pathology. Plasma p-tau231 and p-tau205 may additionally function as markers for different stages of the disease.

**Supplementary Information:**

The online version contains supplementary material available at 10.1007/s00401-023-02660-3.

## Introduction

Alzheimer’s disease (AD) neuropathological changes include the intracellular accumulation of hyperphosphorylated tau (p-tau) as neurofibrillary tangles (NFTs) and neuropil threads as well as the aggregation of extracellular amyloid beta (Aβ) plaques [17]. Accurate and early detection of AD neuropathological changes during life is critical for timely therapeutic intervention [11]. Analysis of cerebrospinal fluid (CSF) and positron emission tomography (PET) represent gold standard methods for the in vivo detection of AD but are expensive and invasive. Plasma-based assays can now detect various phosphorylation tau sites in blood [9], providing an accessible and cost-effective alternative for disease detection with similar prognostic and diagnostic accuracies [15, 22, 28].

Evaluation of plasma p-tau biomarkers against post-mortem AD pathology is necessary for validating their use for clinical purposes. There are over 80 tau phosphorylation sites that can be abnormally phosphorylated during AD progression [27]. Among these, plasma p-tau181, p-tau217, and p-tau231 have been the most studied due to the availability of specific immunoassays for their quantification. Higher levels of plasma p-tau181 have been associated with increased odds for having autopsy-confirmed AD [20], and to discriminate between AD and non-AD pathology up to 8 years prior to death [13]. Plasma p-tau217 can distinguish between individuals with autopsy-confirmed AD and cognitively unimpaired individuals with better accuracy than alternative plasma and MRI-based measures of AD pathology [21], and has been shown to reflect both amyloid and tau pathologies [14]. Plasma p-tau231 is the earliest blood tau biomarker to increase in relation to AD neuropathological changes [1] and is selectively associated with amyloid plaques [2]. Only two plasma-to-autopsy studies conducting head-to-head comparisons of p-tau epitopes for the detection of AD neuropathology have been published [24, 25], but they examine two to three p-tau variants using immunoassays, which require separate sample preparation and analysis for each targeted epitope. Mass spectrometry (MS) allows for increased specificity for detection of low abundant proteins and can quantify numerous p-tau epitopes simultaneously in a single acquisition [3, 18]. This enables the characterization of the specific sites abnormally phosphorylated at each stage of the disease with a fair head-to-head comparison, providing the information for an effective utilization of blood tau biomarkers in both clinical practice and therapeutic trials.

Here, we used an IP-MS method to examine the association between six p-tau (p-tau181, p-tau199, p-tau202, p-tau205, p-tau217, p-tau231) and two non-phosphorylated (tau195–205, tau212–221) tau species in plasma ante-mortem samples and post-mortem AD neuropathological examination. We compared the accuracy of each tau species for discriminating brain donors with and without autopsy-confirmed AD, including among those with and without dementia during life. Each tau form was examined against Braak staging for NFTs, CERAD neuritic amyloid plaque score, and semi-quantitative ratings of p-tau severity across seven different cortical and subcortical brain regions.

## Materials and methods

### Study design and brain donors

Participants were 123 brain donors from the National Institute on Aging (NIA)-funded Boston University Alzheimer’s Disease Research Center (BU ADRC) Neuropathology Core who had ante-mortem blood draw as part of their participation in the BU ADRC Clinical Core. The BU ADRC follows older adults from the Greater Boston area. Participants are older adults with adequate visual acuity and hearing. Participants are excluded for conditions or disorders that interfere with making accurate neurodegenerative disease diagnoses and/or preclude study participations (e.g., serious mental illness, brain tumor, multiple sclerosis, and unstable medical conditions). Procedures involve an annual National Alzheimer’s Coordinating Center Uniform Data Set evaluation. Participants are asked to donate their brain following death to the BU ADRC brain bank for neuropathological processing and examination. Voluntary annual blood draws began at the BU ADRC in 2008 [8, 20, 26]. The current sample set included all participants who had an ante-mortem plasma sample and who donated their brain for neuropathological examination at the time of this study (2022). If multiple blood draws were performed, the sample proximate to death was used. Procedures including brain donation were approved by the BU Medical Campus Institutional Review Board. Participants (or their Legally Authorized Representatives) provided written informed consent prior to participation in the BU ADRC protocol. Next of kin provided written informed consent if written informed consent from the participant was obtained more than 3 years prior to death.

### Plasma collection and biomarker quantification by mass spectrometry (MS)

Blood collection, processing, and storage followed standard operating procedures that adhere to the National Centralized Repository for AD and Related Dementias. Non-fasting blood samples were collected into plastic dipotassium EDTA tubes. Sample were processed with plasma aliquoted and frozen at − 80 °C. Frozen plasma aliquots were shipped on dry ice to the University of Gothenburg (Sweden) for batch analysis. The in-house MS method was previously described [18] and was used to analyze 1-ml EDTA plasma samples. For further details, see Supplementary Methods and Supplementary Tables 1 and 2.

### Neuropathological evaluation

Neuropathological processing and evaluation were conducted using published methods [29, 30]. Procedures followed the National Alzheimer’s Coordinating Center standardized Neuropathology Form and Coding Guidebook [4, 5, 16]. The neuropathological diagnosis of AD was made using the NIA-Reagan Institute criteria. The AD group was defined by brain donors who had intermediate or high likelihood of AD. Published criteria were used for neuropathological diagnoses of other neurodegenerative diseases [7]. Neuropathologists used semi-quantitative scales (0 [none]–3 [severe]) to rate severity of cerebral amyloid angiopathy, atherosclerosis, and arteriolosclerosis. The Consortium to Establish a Registry for Alzheimer’s Disease (CERAD) score was used to evaluate the presence and severity of neuritic Aβ plaques. Braak staging of NFTs was rated on a scale from 0 (no NFTs) to VI (widespread NFTs with marked involvement of the neocortex). In addition to Braak scores, neuropathologists rated the density of p-tau pathology in various cortical and subcortical regions using semi-quantitative rating scales (0 [none]–3 [severe]). AT8-immunostained, 10 µm thick paraffin-embedded sections of the regions affected in AD were examined and included: dorsolateral frontal cortex, inferior orbital frontal cortex, superior temporal cortex, inferior parietal cortex, CA1-hippocampus, CA2-hippocampus, CA4-hippocampus, entorhinal cortex, amygdala, and locus coeruleus. Frontal and hippocampal regions were combined to form a frontal cortex and hippocampus composite, respectively.

### Dementia severity

The Clinical Dementia Rating (CDR) Dementia Staging Instrument was used to evaluate dementia severity at the time of the blood draw [19].

### Statistical analysis

The primary predictors included the six p-tau (p-tau181, p-tau199, p-tau202, p-tau205, p-tau217, and p-tau231) and the two non-phosphorylated peptides (tau195–209 and tau212–221). We examined the following ratios: p-tau217/tau212–221 and p-tau205/tau195–209 because both the phosphorylated and the non-phosphorylated forms comprising the same amino acid sequence are included in the panel. All tau variables were standardized as z-scores. Outcomes included AD status (yes/no), CERAD score, Braak score, and the semi-quantitative ratings of regional p-tau (frontal cortex, superior temporal, inferior parietal, entorhinal cortex, amygdala, hippocampus, and locus coeruleus). Binary logistic regression models examined the association between each tau epitope and AD status. For each model, discrimination accuracy for AD neuropathological diagnosis was evaluated using the area under the receiver operating characteristic curve (AUC) statistic based on the predicted probabilities from the multivariable logistic regression that included the relevant covariates (see below). Discrimination accuracy was categorized based on guidelines suggested in Hosmer and Lemeshow [10]. For sensitivity analyses, we repeated models stratified by CDR scores at the time of blood draw (< 1 and ≥ 1). In the entire sample, multivariable ordinal logistic regressions tested the associations between each plasma tau variant and Braak NFT stage (stage 0, I/II, III/IV, V/VI) and CERAD neuritic plaque score. Linear regressions were used for the ratings of p-tau severity in each cortical and subcortical brain region. Sample size for the semi-quantitative ratings of regional p-tau severity was reduced due to missingness. Bivariate Pearson’s correlations tested the associations between plasma and brain p-tau variants (i.e., p-tau181, p-tau202, and p-tau231). Model covariates included age at death, years between last blood draw and death, sex (1 = female, 0 = male), and *APOE* ε4 status (1 = ε4 carrier, 0 = non-carrier). All analyses were conducted using R software. A *P *value < 0.05 was considered statistically significant.

### Data availability

All uniform and neuropathology data set evaluation data are shared with the National Alzheimer’s Coordinating Center and are publicly available. Data are available upon reasonable request to the BU ADRC.

### Role of the funding source

The funders of the study had no role in study design, data collection, data analysis, data interpretation, or writing of the report.

## Results

### Sample characteristics

Table 1 and Supplementary Table 3 present sample characteristics, stratified by neuropathological AD status. The mean (standard deviation) time between blood draw and death was 5.5 (3.13) years with a median of 5.0 and range of 0.0 (blood draw done same month of death)–12.0 years. Sixty-nine (56.1%) had AD at autopsy. Compared with those without AD, those with AD were more likely to have an *APOE* ε4 allele (*P* = 0.01) and a higher global CDR score at time of blood draw and death (*P* < 0.01).

**Table 1:** Sample characteristics

	Total sample set (*N* = 123)	AD (*N* = 69)	Non-AD (*N* = 54)	*P* value
Demographics				
Sex, *n* (%) female	55 (44.7)	29 (42.0)	26 (48.1)	0.50
Age at blood draw, mean (SD)	78.17 (8.55)	77.23 (8.96)	79.37 (7.91)	0.17
Age at death, mean (SD)	83.71 (8.92)	82.30 (8.96)	85.50 (8.62)	0.05
Race, *n* (%)				0.24
American Indian/Alaska Native	2 (1.6)	2 (2.9)	0	
Asian	1 (0.8)	0	1 (1.9)	
Black or African American	6 (4.9)	2 (2.9)	4 (7.4)	
White	113 (91.9)	65 (94.2)	48 (88.9)	
Other	1 (0.8)	0	1 (1.9)	
Diagnosis at death, *n* (%)				< 0.01
Normal cognition	15 (12.3)	0 (0)	15 (27.8)	
MCI/non-MCI cognitively impaired	25 (20.5)	7 (10.3)	18 (33.3)	
Dementia	82 (67.2)	61 (89.7)	21 (38.9)	
Dementia severity				
Global CDR score at death, *n* (%) < 1	69 (56.1)	25 (36.2)	44 (81.5)	< 0.01
≥ 1	54 (43.9)	44 (63.8)	10 (18.5)	
Global CDR score at blood draw, *n* (%) < 1	71 (57.7)	26 (37.7)	45 (83.3)	< 0.01
≥ 1	52 (42.3)	43 (62.3)	9 (16.7)	
Genetic				
*APOE ε* 4 allele status, *n* (%) carrier	53 (44.2)	37 (54.4)	16 (30.8)	0.01
Comorbidities				
Hypertension	59 (48.0)	29 (42.0)	30 (55.6)	0.14
Diabetes	14 (11.4)	9 (13.0)	5 (9.3)	0.58
Sleep apnea	10 (8.1)	4 (5.8)	6 (11.1)	0.33
Plasma biomarker, mean (SD)/range, (fmol/ml)				
P-tau181	0.06 (0.03)/0.01–0.18	0.07 (0.03)/0.03–0.18	0.05 (0.02)/0.01–0.14	< 0.01
P-tau199	0.01 (0.003)/0.00–0.02	0.01 (0.003)/0.00–0.02	0.01 (0.00)/0.00–0.02	< 0.01
P-tau202	0.01 (0.004)/0.00–0.03	0.01 (0.005)/0.00–0.03	0.01 (0.003)/0.00–0.02	0.03
P-tau205	0.00 (0.00)/0.00–0.00	0.00 (0.000)/0.00–0.00	0.000 (0.000)/0.00–0.00	< 0.01
P-tau217	0.01 (0.01)/0.01–1.11	0.02 (0.01)/0.00–0.07	0.01 (0.005)/0.00–0.02	< 0.01
P-tau231	0.03 (0.03)/0.00–0.12	0.04 (0.02)/0.01–0.12	0.02 (0.01)/0.00–0.08	< 0.01
Tau195–209	0.65 (0.26)/0.01–1.61	0.73 (0.28)/0.02–1.61	0.55 (0.19)/0.01–1.22	< 0.01
Tau212–221	0.49 (0.17)/0.01–1.11	0.53 (0.18)/0.18–1.11	0.44 (0.15)/0.01–0.99	< 0.01
P-tau217/tau212–221	0.03 (0.02)/0.01–0.07	0.04 (0.01)/0.01–0.07	0.01 (0.01)/0.01–0.04	< 0.01
P-tau205/tau195–209	0.00 (0.00)/0.00–0.00	0.00 (0.00)/0.00–0.00	0.00 (0.00)/0.00–0.00	< 0.01

Binary logistic regression compared donors with and without pathological Alzheimer’s disease on binary outcomes; independent samples t-test was used for continuous outcomes. White and non-white were compared (1 = white, 0 = non-white). Sex was coded as 0 (male) and 1 (female). Sample sizes: diagnosis at death, *n* = 122 due to missingness; *APOE ε* 4 allele status, *n* = 120 due to missingness; plasma biomarkers, *n* = 121 due to technical errors

*AD* Alzheimer’s disease, *CDR* clinical dementia rating (CDR) dementia staging instrument, *MCI* mild cognitive impairment

Brain donors with autopsy-confirmed AD had more severe cerebral amyloid angiopathy and regional p-tau than non-AD (*P*-values < 0.01). There were no other statistically significant differences between AD and non-AD in neuropathological diagnoses.

### Plasma tau variants in brain donors stratified by autopsy-confirmed AD status

**Fig. 1:**
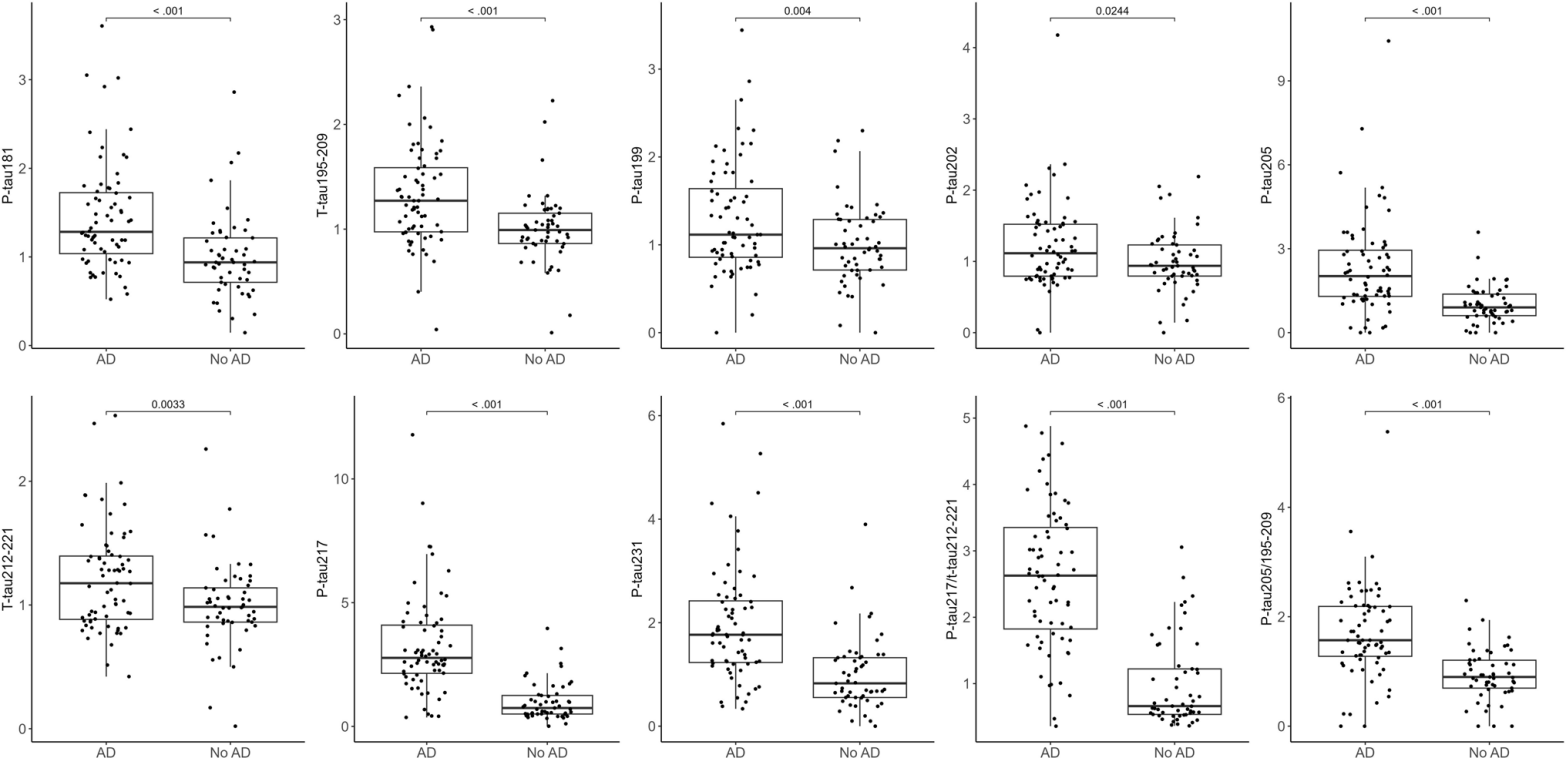
Box plots of the fold-changes of the tau peptide concentrations by Alzheimer’s disease status. The non-AD group was used as a reference. National Institute on Aging-Reagan Institute criteria were used for the neuropathological diagnosis of Alzheimer’s disease. Box plots include the median (bar) and interquartile range (whiskers) as well as the individual data points

Figure 1 shows the distribution of plasma tau epitope concentrations by AD status represented as fold-changes (FC) of the mean using the control group as a reference. The measured raw concentrations for each tau species are depicted in Supplementary Fig. 1. Plasma p-tau205, p-tau217 and p-tau231 showed highest increases in AD compared with controls (FC_p-tau205_ = 2.02, FC_p-tau217_ = 2.77, FC_p-tau231_ = 1.77, *P* < 0.001 in all cases). These FC were not higher when using the ratio phosphorylated/non-phosphorylated peptide for p-tau205 and p-tau217 (FC_p-tau205/t-tau195–209_ = 1.57, FC_p-tau217/t-tau212–221_ = 2.63). Plasma p-tau181, p-tau199, p-tau202, and the non-phosphorylated peptides 195–209 and 212–221 showed moderate yet significant fold-changes (FC_p-tau181_ = 1.28, *P* < 0.001; FC_p-tau199_ = 1.12, p = 0.004; FC_p-tau202_ = 1.11, *P* = 0.0244; FC_tau195–209_ = 1.27, *P* < 0.001; FC_tau212–221_ = 1.18, *P* = 0.033). Binary logistic regressions (Table 2) showed that higher levels of each plasma p-tau and tau form were associated with increased odds for having AD (ORs = 1.70 [p-tau202]—27.00 [p-tau217]), which were particularly high for p-tau217 (OR = 27.00, CI 95% (8.6–112.33)) and the p-tau217/tau212–221 ratio (OR = 11.03, CI 95% (4.93–30.42)). The plasma p-tau217/tau212–221 ratio and p-tau217 had outstanding accuracy for discriminating brain donors with AD from those without AD (AUC_p-tau217/tau212–221_ = 90.0, 95% CI = 84.1–96.0; AUC_p-tau217_ = 89.8, 95% CI = 83.8–95.8). Epitopes with excellent discrimination accuracy included plasma p-tau231 (AUC = 83.4, 95% CI = 75.6–91.2), p-tau205/tau195–209 ratio (AUC = 82.1, 95% CI = 73.9–90.2), and p-tau205 (AUC = 81.3, 95% CI = 73.2–89.4). Plasma p-tau199 (AUC = 72.1, 95% CI = 73.2–89.4), p-tau202 (AUC = 71.1, 95% CI = 73.2–89.4), tau195–209 (AUC = 78.1, 95% CI = 62.4–81.7) and tau212–221 (AUC = 72.6, 95% CI = 63.1–82.1) showed acceptable discrimination accuracy.

**Table 2:** Associations between plasma tau species and Alzheimer’s disease neuropathology

	OR (95% CI)	AUC (95% CI)
Total sample: 69 AD, 54 no AD		
P-tau217/tau212–221	11.03 (4.93, 30.42)	90.0 (84.1, 96.0)
P-tau217	27.00 (8.60, 112.33)	89.8 (83.8, 95.8)
P-tau231	5.28 (2.61, 12.43)	83.4 (75.6, 91.2)
P-tau205/tau195–209	4.07 (2.21, 8.49)	82.1 (73.9, 90.2)
P-tau205	5.62 (2.57, 14.60)	81.3 (73.2, 89.4)
P-tau181	3.02 (1.74, 5.84)	79.0 (70.3, 87.6)
Tau195–209	2.47 (1.47, 4.66)	78.1 (69.3, 86.9)
Tau212–221	1.85 (1.18, 3.15)	72.6 (63.1, 82.1)
P-tau199	1.83 (1.15, 3.11)	72.1 (62.4, 81.7)
P-tau202	1.70 (1.05, 2.91)	71.1 (61.3, 80.9)
CDR < 1.0: 26 AD, 45 no AD		
P-tau217	22.04 (5.57, 137.05)	86.3 (76.7, 96.0)
P-tau217/tau212–221	8.34 (3.31, 27.35)	85.6 (75.1, 95.9)
P-tau231	5.13 (2.17, 15.20)	80.6 (69.3, 91.9)
P-tau205	7.06 (2.28, 27.98)	77.1 (64.8, 89.4)
P-tau205/tau195–209	4.74 (1.79, 20.10)	76.5 (63.5, 90.0)
P-tau181	2.37 (1.28, 4.94)	71.3 (58.6, 84.0)
Tau195–209	2.65 (1.26, 6.67)	72.8 (59.5, 86.1)
Tau212–221	1.65 (0.89, 3.39)	64.0 (49.6, 78.4)
P-tau199	1.65 (0.82, 3.56)	63.3 (48.6, 78.0)
P-tau202	1.50 (0.76, 3.09)	63.3 (48.7, 77.9)
CDR ≥ 1.0: 43 AD, 9 no AD		
P-tau217	189.34 (9.76, 26,020.65)	96.1 (90.7, 100.0)
P-tau217/tau212–221	54.82 (5.32, 5625.03)	96.1 (90.1, 100.0)
P-tau231	19.96 (2.69, 691.77)	92.2 (84.3, 100.0)
P-tau205/tau195–209	4.74 (1.79, 20.08)	91.9 (83.8, 100.0)
P-tau205	5.66 (1.68, 36.66)	90.1 (81.1, 99.1)
P-tau181	17.42 (2.75, 372.20)	89.2 (78.7, 99.7)
Tau195–209	2.12 (0.97, 6.31)	84.4 (72.7, 96.0)
Tau212–221	2.63 (1.07, 9.39)	81.7 (69.1, 94.3)
P-tau199	2.03 (0.97, 5.65)	80.2 (66.4, 94.0)
P-tau202	2.17 (0.91, 6.92)	81.1 (68.4, 93.7)

Binary logistic regression examined the association between plasma tau levels and intermediate to high Alzheimer’s disease neuropathologic change (per NIA-Reagan criteria). Models controlled for age at death, years between last blood draw and death, sex, and *APOE* ε4 carrier status. The AUC statistics were calculated using predicted probabilities from the binary logistic regression. Biomarkers are presented in rank order of their AUC statistic

*AD* Alzheimer’s disease, *CDR* clinical dementia rating (CDR) dementia staging instrument, *OR* odds ratio, *CI* confidence interval, *p-tau* phosphorylated tau

### Plasma tau variants in brain donors stratified by global CDR score at blood draw

Corresponding to global CDR scores at time of blood draw, there were 71 brain donors (57.7%) with CDR < 1 and 52 (42.3%) with CDR ≥ 1 (Supplementary Figs. 2 and 3). For all epitopes, there was better discrimination accuracy among those with high CDR scores compared with low (Table 2). AUCs ranged from 80.2 (p-tau199) to 96.1 (p-tau217, p-tau217/tau212–221 ratio) among those with a CDR ≥ 1.0. Among those with a CDR < 1.0, there was still excellent discrimination accuracy for p-tau217 (AUC = 86.0), p-tau217/tau212–221 ratio (AUC = 86.0), and p-tau231 (AUC = 80.1) as well as acceptable discrimination accuracy for p-tau205 (AUC = 77.1), p-tau205/tau195–209 ratio (AUC = 76.5), tau195–209 (AUC = 72.8), and p-tau181 (AUC = 71.0). However, AUCs fell below 0.70 (not acceptable discrimination) for p-tau199, p-tau202, and tau212–221.

### Associations between plasma tau variants and autopsy rating scales of amyloid pathology

As shown in Table 3, ordinal logistic regressions showed that higher concentrations of all tau peptides were associated with CERAD neuritic plaque score. The effect sizes were strongest for p-tau217 (OR = 15.24, 95% CI = 6.72–34.53) followed by the ratio of p-tau217/tau212–221 (OR = 7.62, 95% CI = 4.26–13.65), p-tau205 (OR = 5.05, 95% CI = 2.65–9.62) and p-tau231 (OR = 3.86, 95% CI = 2.26–6.62)). Plasma p-tau199 and p-tau202, together with tau195–209 and tau212–221 showed moderate, yet significant associations.

**Table 3:** Associations between plasma tau species and Braak stage and CERAD neuritic amyloid plaque score

	OR (95% CI)	*P* value
Braak staging for NFTs		
P-tau217	14.29 (5.71, 35.79)	< 0.01
P-tau217/tau212–221	8.88 (4.41, 17.89)	< 0.01
P-tau205	5.27 (2.54, 10.94)	< 0.01
P-tau205/tau195–209	4.52 (2.52, 8.11)	< 0.01
P-tau231	4.02 (2.20, 7.32)	< 0.01
P-tau181	2.53 (1.56, 4.11)	< 0.01
Tau195–209	1.98 (1.26, 3.10)	< 0.01
P-tau199	1.76 (1.14, 2.70)	0.01
Tau212–221	1.67 (1.11, 2.51)	0.01
P-tau202	1.61 (1.04, 2.50)	0.03
CERAD neuritic amyloid plaque score		
P-tau217	15.24 (6.72, 34.53)	< 0.01
P-tau217/tau212–221	7.62 (4.26, 13.65)	< 0.01
P-tau205	5.05 (2.65, 9.62)	< 0.01
P-tau231	3.86 (2.26, 6.62)	< 0.01
P-tau205/tau195–209	3.17 (1.95, 5.17)	< 0.01
P-tau181	2.52 (1.62, 3.91)	< 0.01
Tau195–209	2.37 (1.51, 3.71)	< 0.01
P-tau199	2.21 (1.42, 3.44)	< 0.01
Tau212–221	1.97 (1.31, 2.96)	< 0.01
P-tau202	1.94 (1.22, 3.07)	0.01

Multivariable ordinal logistic regression examined the association between plasma tau levels and Braak staging for NFTs (0, I/II, III/IV, V/VI) and CERAD neuritic amyloid plaque score. Models controlled for age at death, years between last blood draw and death, sex, and *APOE* ε4 carrier status. Presented in rank order of effect size

*OR* odds ratio, *CI* confidence interval, *p-tau* phosphorylated tau

**Fig. 2:**
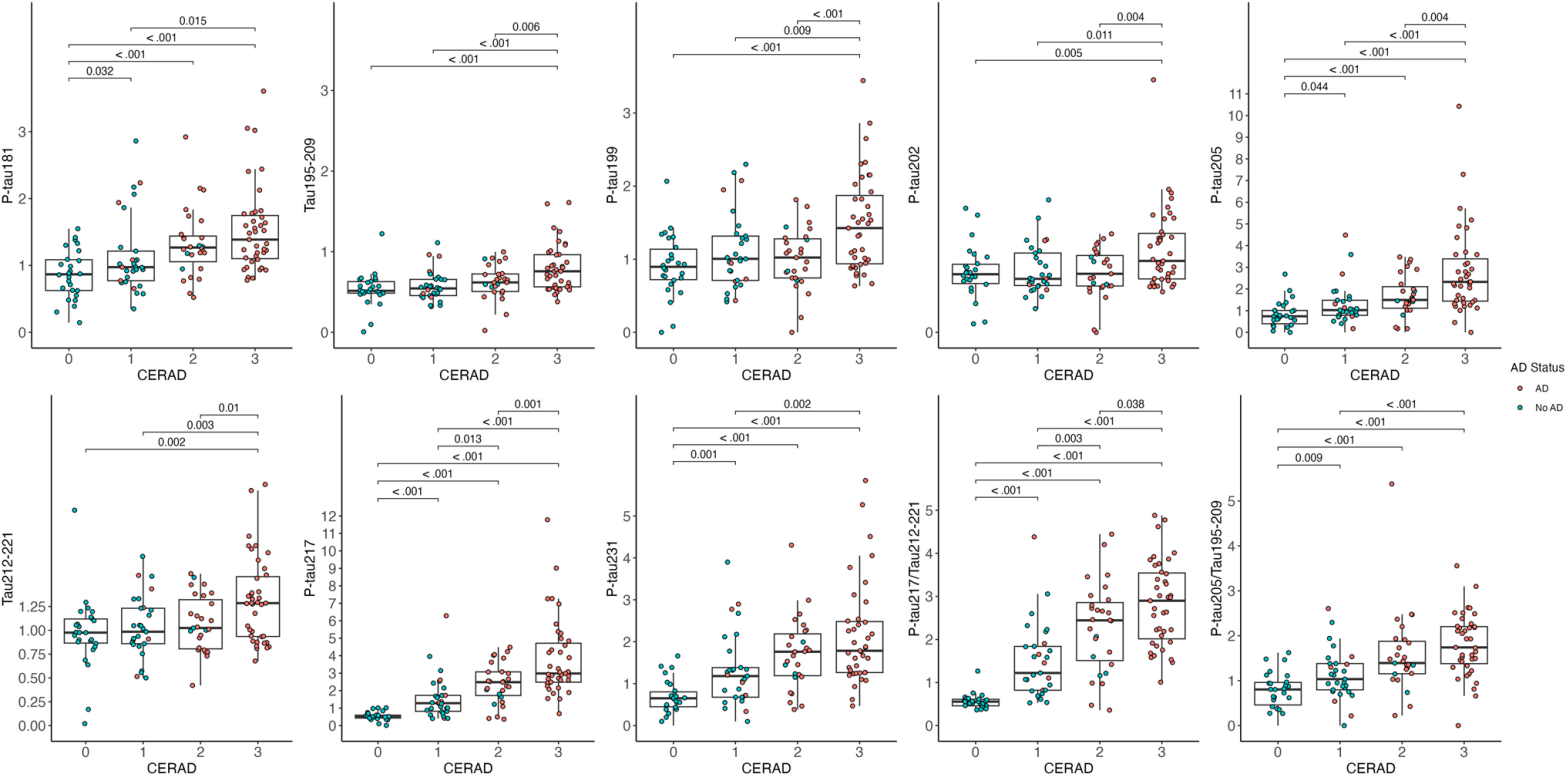
Box plots of the fold-changes of the tau peptide concentrations by CERAD neuritic amyloid plaque score. CERAD 0 was used as the reference group. Box plots include the median (bar) and interquartile range (whiskers) as well as the individual data points**.** Participants are color-coded based on the presence (red) or absence (blue) of AD brain pathology

Figure 2 represents the fold-changes in the levels of the plasma tau biomarkers when brain donors were grouped by CERAD scores. The raw concentrations for each tau species are depicted in Supplementary Fig. 4. P-tau205, p-tau217, and p-tau231 were the variants with higher fold-changes with advancing disease, but with subtle differences. Plasma p-tau231 showed the most pronounced increases from CERAD 0 to 1 (FC = 2.3) and plateaued between scores 2 and 3 (FC = 1). Plasma p-tau217 and the p-tau217/212–221 ratio presented the highest fold-changes between CERAD 1 and 2 (FC_p-tau217_ = 1.9 and FC_p-tau217/tau212–221_ = 2.0). Plasma p-tau205’s most pronounced increase was observed from CERAD 2 to 3 (FC = 1.6), but interestingly, this was not conserved for the p-tau205/tau195–209 ratio, which showed the largest change from stage 1 to 2 (FC = 1.39). Plasma p-tau199, p-tau202 and the non-phosphorylated tau195–209 and tau212–221 presented moderate increases, but in all the cases, significant between CERAD 2 and 3.

### Association between plasma tau variants and autopsy rating scales of tau pathology

**Fig. 3:**
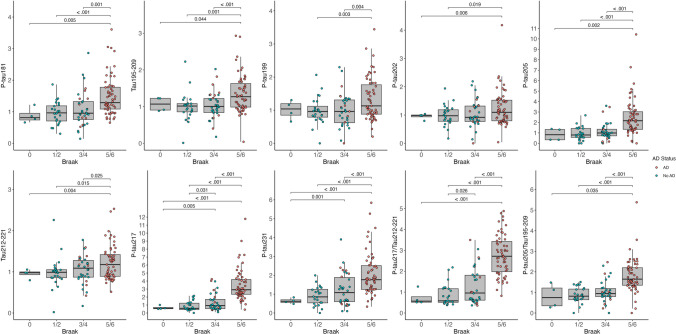
Box plots of the fold-changes of the tau peptide concentrations by Braak staging for NFTs. Braak I–II was used as the reference group. Box plots include the median (bar) and interquartile range (whiskers) as well as the individual data points. Participants are color-coded based on the presence (red) or absence (blue) of AD brain pathology

Ordinal logistic regressions between Braak stages and the levels of the different tau peptides (Table 3) indicated that the effect sizes were strongest for p-tau217 (OR = 14.29, 95% CI = 5.71–35.79)) followed by the ratio of p-tau217/tau212–221 (OR = 8.88, 95% CI = 4.41–17.89), p-tau205 (OR = 5.27, 95% CI = 2.54–10.94), the ratio of p-tau205/tau195–209 (OR = 4.52, 95% CI = 2.52–8.11)) and then p-tau231 (OR = 4.02, 95% CI = 2.20–7.32). When brain donors were grouped by Braak stages (Fig. 3 and Supplementary Fig. 5), p-tau231 presented the earliest significant changes at Braak III–IV. Plasma p-tau217 showed the earliest significant increases from Braak III–IV but had further higher levels in Braak V–VI; plasma p-tau205 changes were only significant in Braak V–VI. Similar to the findings with CERAD, p-tau199, p-tau202 and the non-phosphorylated peptides tau195–209 and tau212–221 presented moderate increases, but in all cases were significant at late stages of the disease, i.e., Braak V–VI. None of the plasma tau biomarkers were increased in primary age-related tauopathy (PART) (CERAD 0, Braak III–IV), compared to controls (CERAD 0, Braak 0–II) (Supplementary Fig. 6). The levels of p-tau217, p-tau231, and the ratio p-tau217/tau212–221 were increased in AD cases (CERAD ≥ 1) compared to PART with the same tau burden (Braak III–IV). All biomarkers were significantly higher in advanced AD (CERAD ≥ 1, Braak V–VI) compared to PART.

Correlations with regional p-tau severity at autopsy (Supplementary Table 4) showed that plasma p-tau217 and the ratio of p-tau217/tau212–221 consistently had the strongest associations with p-tau severity across many different cortical and subcortical brain regions (standardized betas p-tau217 = 0.49 [amygdala]–0.68 [frontal cortex]; standardized betas p-tau217/tau212–221 = 0.58 [entorhinal cortex, locus coeruleus]–0.77 [frontal cortex]). Plasma p-tau205, the ratio of p-tau205/tau195–209, and p-tau231 also demonstrated consistent and modest associations with p-tau severity ratings, followed by p-tau181 and p-tau199. Plasma p-tau202, tau195–209, and tau212–221 had weak associations with the p-tau severity ratings.

## Discussion

A multitude of studies have demonstrated the association of plasma p-tau with amyloid and tau PET imaging, CSF biomarkers, and cognition, but relatively few have described the relation to autopsy findings. Such studies report results from a single phosphorylation site, and rarely compare multiple p-taus. Here, we determine for the first time, the levels of six phosphorylated and two non-phosphorylated tau species simultaneously quantified by MS in ante-mortem plasma of brain donors. We found that the concentrations of all p-tau and non-phosphorylated tau peptides were increased in neuropathologically confirmed AD, but p-tau217, p-tau205, and p-tau231 were the species with larger dynamic ranges. In particular, p-tau217 was the most accurate biomarker discriminating brain donors by AD and cognitive status and showed the highest associations with amyloid and tau neuropathological ratings. However, evaluation of the levels of the tau peptides with CERAD and Braak classifications indicated that different phosphorylated tau species increase at different stages of the disease. Taken together, these results contribute to the existing literature by not only demonstrating the capability of plasma p-tau to detect AD pathology but also revealing distinctions among p-tau species. We postulate that these differences will be valuable for selecting the most suitable biomarker in different scenarios, i.e., diagnosis, prognosis, or treatment monitoring.

By directly comparing the levels of the tau biomarkers in plasma, we observed that p-tau217, followed by p-tau205 and p-tau231, exhibited the highest fold-changes in AD cases compared with non-AD cases, greater than the other p-tau (181, 199, 202) and non-phosphorylated tau (195–209, 212–221). Those peptides with the largest dynamic ranges also had the greatest capacity to discriminate between neuropathologically confirmed AD and non-AD participants. Plasma p-tau217 was the biomarker with superior performance, followed by p-tau231 and p-tau205, which had similar accuracies. Normalization of p-tau217 and p-tau205 concentrations with their respective non-phosphorylated peptides (p-tau217/212–221 and p-tau205/195–209) rendered a similar accuracy, in concordance [18] and contrast [3] to previous work. When we analyzed the performance of the different tau species to detect AD when brain donors were stratified by dementia status at the time of the blood draw, all biomarkers showed a superior prediction in demented participants (CDR ≥ 1). This is probably due to higher tau levels in blood with more advanced disease. However, the accuracy in those with normal cognition or mild cognitive impairment (CDR < 1) remained high for most tau peptides. The same pattern as neuropathological examination was observed: p-tau217 was the biomarker with highest performance, followed by p-tau205 and p-tau231. These results are in line with previous studies showing that among the currently available plasma biomarkers, p-tau217 concentrations reflect underlying AD pathology with the greatest fidelity as determined by neuropathology [14, 24], PET imaging [21], and CSF biomarkers [12]. Longitudinally, p-tau217 has been shown as the only available blood biomarker with marked amyloid-dependent changes and with increases associated with clinical deterioration and brain atrophy [1]. These studies encompass comparisons with other tau phopshorylations, such as p-tau181 or p-tau231, but not with p-tau205 due to the previous lack of an available assay.

Through the utilization of our MS method, we recently described that plasma p-tau231, p-tau217, and p-tau205 exhibited stronger correlations with PET signals compared to other tau species, but their variations were associated differently with amyloid and tau PET [18]. P-tau231 was influenced more by amyloid, p-tau217 by both amyloid and tau, and p-tau205 primarily by tau. Here, we investigated associations between the plasma tau species and neuropathological scores of amyloid (CERAD) and tau (Braak) accumulation in the brain. We also observed that p-tau217, p-tau231, and p-tau205 displayed higher associations with the neuropathological staging of amyloid and tau, as well as with p-tau severity ratings at autopsy. Interestingly, plasma p-tau231 exhibited the most significant fold-changes with mild β-amyloid plaque density and reached a plateau between moderate and severe scores. Plasma p-tau217 showed the highest raise with moderate amyloid plaque density and at Braak III–IV, and continually increased thereafter. Meanwhile, plasma p-tau205 levels changed the most with severe amyloid plaque score and in Braak V–VI. The remaining peptides displayed their most pronounced changes—although moderate—in late disease stages (CERAD 3 and Braak V–VI). This observation suggests a widespread increase in phosphorylation and overall tau levels as the disease advances significantly.

These findings emphasize that not all tau markers are equal or indicative of the same brain changes. Among them, p-tau217 emerges as the most promising biomarker, exhibiting higher dynamic ranges and superior accuracy. However, other markers can provide valuable insights into different underlying processes. In the successful TRIALBLAZER-ALZ 2 clinical trial, participants were recruited based on their tau PET burden [23], showing the best results in low-medium tau PET population. The plasma p-tau profile characterization of each patient with a method like the one presented herein could assist in defining the target participants for a specific AD treatment.

There are limitations to the current findings. First, we did not explore trends in plasma tau species levels longitudinally. While plasma tau peptides accurately detect AD at autopsy, the clinical significance of a unit increase in raw values remains unclear. In addition, the findings are limited to participants from a single clinical cohort, which introduces the potential for selection bias. The present sample is from a National Institute on Aging-funded ADRC and is most representative of individuals who present to a clinic with concerns regarding their cognitive functioning. This population allows for development and validation of biomarkers, but inferences regarding risk and screening for AD in the general population cannot be made. Furthermore, the sample exhibited demographic homogeneity, with a majority identifying as white. This homogeneity could limit the broader applicability of the results to more diverse populations. We did not include the examination of other potential clinical, genetic, demographic, and social determinants of health variables and how they relate to the studied plasma biomarkers. Prospective population-based studies are warranted to address these knowledge gaps and identify cutoff values that optimize sensitivity and specificity for the detection of AD in a wider range of individuals. The AUC values reported in the present work appear lower than studies analyzing blood-based biomarkers against imaging and fluid measures. In most investigations, PET and CSF markers demonstrate an accuracy of 90–95% when compared to post-mortem. Hence, in the context of in vivo research where p-tau is shown to have an AUC > 0.95, this evaluation is conducted against a “gold standard” that is not precise. Thus, the likely explanation for the AUC values being less than 0.90 in this study is rooted in the evaluation against actual neuropathology. In addition, we are comparing blood biomarkers to end-stage disease—which is an unlikely scenario (e.g., it will be a preclinic or MCI stage). Future studies should address if the overlap cases between AD and non-AD groups would benefit from a two-step diagnostic workflow, as recently proposed [6]. In that case, blood tau biomarkers could serve as a first screening tool for AD pathology (step 1), followed by confirmatory testing with CSF Aβ42/Aβ40 or PET imaging (step 2) only in patients with intermediate risk at the first step. This would reduce the need for confirmatory testing while accurately classifying patients, offering a viable option to centers that do not have access to specialized testing. Lastly, we recognize that while MS techniques provide the potential for multiplexing various phosphorylations, offering a distinct platform for AD staging, they demand more extensive efforts for scalability and implementation in clinical routine than other platforms.

In summary, our findings support plasma p-tau217 as the most promising p-tau species for detecting AD brain pathology. Plasma p-tau231 and p-tau205 may additionally function as markers for different stages of the disease.

### Supplementary Information

Below is the link to the electronic supplementary material.

Supplementary file1 (DOCX 1518 KB)
