# Supplementary material for: Optimal blood tau species for the detection of Alzheimer’s disease neuropathology: an immunoprecipitation mass spectrometry and autopsy study
Source: Acta Neuropathol. 2023 Dec 30;147(1):5. doi: 10.1007/s00401-023-02660-3 (PMC10757700; doi:10.1007/s00401-023-02660-3)
Supplement: Supplementary file 1 — Supplementary file1 (DOCX 1518 KB) [file 401_2023_2660_MOESM1_ESM.docx]

**Optimal Blood Tau Species for the Detection of Alzheimer’s Disease Neuropathology: An Immunoprecipitation Mass Spectrometry and Autopsy Study**

Laia Montoliu-Gaya*,^1^ Michael L. Alosco*,^2,3^ Euky Yhang,^4^ Yorghos Tripodis,^4^ Daniel Sconzo,^2^ Madeline Ally,^5^ Lana Grötschel^1^ , Nicholas J. Ashton^1, 6, 7, 8^,^,^ Juan Lantero-Rodriguez^1^, Mathias Sauer^1^, Bárbara Gomes^1^, Johanna Nilsson^1^, Gunnar Brinkmalm^1^, Michael A. Sugarman^9^, Hugo J. Aparicio,^2,3^ Brett Martin^10^, Joseph N. Palmisano^10^, Eric G. Steinberg,^2,3^ Irene Simkin^11^, Katherine W. Turk,^2,3,12^ Andrew E. Budson,^2,3,12^ Rhoda Au^2,3,11,13^, Lindsay Farrer^2,3,11,13^, Gyungah R. Jun^2,11^, Neil W. Kowall^2,3^, Robert A. Stern, PhD,^2,3,14,15^ Lee E. Goldstein, ^2,3,16,17^ Wei Qiao Qiu,^2,3,18,19^ Jesse Mez,^2,3^ Bertrand Russell Huber, MD, PhD^2,3,12^, Victor E. Alvarez, MD^2,3,12,20^, Ann C. McKee^2,3,12,16,21^, Henrik Zetterberg^1, 22, 23, 24, 25, 26^, Johan Gobom^1^, Thor D. Stein^2,14,16,23**^, and Kaj Blennow^1,22**^

*Denotes shared first authors

**Denotes shared senior authors

**Author affiliations:**

^1^Department of Psychiatry and Neurochemistry, Institute of Neuroscience & Physiology, The Sahlgrenska Academy at the University of Gothenburg, Mölndal, Sweden.

^2^Boston University Alzheimer’s Disease Research Center and CTE Center, Boston University Chobanian & Avedisian School of Medicine, Boston, MA 02118, USA

^3^Department of Neurology, Boston University Chobanian & Avedisian School of Medicine, Boston, MA 02118, USA

^4^Department of Biostatistics, Boston University School of Public Health, Boston, MA 02118, USA

^5^University of Arizona, Tucson, AZ

^6^ Centre for Age-Related Medicine, Stavanger University Hospital, Stavanger, Norway.

^7^Department of Old Age Psychiatry, Maurice Wohl Clinical Neuroscience Institute, King’s College London, London, UK.

^8^ NIHR Biomedical Research Centre for Mental Health & Biomedical Research Unit for Dementia at South London & Maudsley NHS Foundation, London, UK.

^9^ Department of Neurology, Medical University of South Carolina, Charleston, SC 29425, USA

^10^Biostatistics and Epidemiology Data Analytics Center, Boston University School of Public Health, Boston, MA 02118, USA

^11^Department of Medicine, Boston University Chobanian & Avedisian School of Medicine, Boston, MA 02118, USA

^12^VA Boston Healthcare System, U.S. Department of Veteran Affairs, Jamaica Plain, MA 02130, USA

^13^Department of Epidemiology, Boston University School of Public Health, Boston, MA 02118, USA

^14^Department of Anatomy & Neurobiology, Boston University School of Medicine, Boston, MA 02118, USA

^15^Department of Neurosurgery, Boston University School of Medicine, Boston, MA 02118, USA

^16^Department of Psychiatry and Ophthalmology, Boston University School of Medicine, Boston, MA 02118, USA

^17^Department of Biomedical, Electrical & Computer Engineering, Boston University College of Engineering, Boston, MA 02215, USA

^18^Department of Pharmacology and Experimental Therapeutics, Boston University Chobanian & Avedisian School of Medicine, Boston, MA 02118, USA

^19^Department of Psychiatry, Boston University Chobanian & Avedisian School of Medicine, Boston, MA 02118, USA

^20^VA Bedford Healthcare System, U.S. Department of Veteran Affairs, Bedford, MA 01730, USA

^21^Department of Pathology and Laboratory Medicine, Boston University School of Medicine, Boston, MA 02118, USA

^22^ Clinical Neurochemistry Laboratory, Sahlgrenska University Hospital, Mölndal, Sweden.

^23^ Department of Neurodegenerative Disease, Queen Square Institute of Neurology, University College London, London, UK.

^24^ UK Dementia Research Institute, University College London, London, UK.

^25^ Hong Kong Center for Neurodegenerative Diseases, Hong Kong, China.

^26^ UW Department of Medicine, School of Medicine and Public Health, Madison, WI, USA

**Correspondence to: laia.montoliu.gaya@gu.se**

**TABLE OF CONTENTS**

[SUPPLEMENTARY METHODS 4](#_Toc149758706)

[Immunoprecipitation Mass Spectrometry Quantification of Tau Peptides 4](#_Toc149758707)

[SUPPLEMENTARY TABLES 5](#_Toc149758708)

[Supplementary Table 1. Tryptic tau peptides targeted in the study. 5](#_Toc149758709)

[Supplementary Table 2. Heavy standards used for the nomalization of quantified peptides. 5](#_Toc149758710)

[Supplementary Table 3. Neuropathology Characteristics. 6](#_Toc149758711)

[Supplementary Table 4. Associations between Plasma Tau Peptides and Ratings of p-Tau Severity at Autopsy 8](#_Toc149758712)

[SUPPLEMENTARY FIGURES 9](#_Toc149758713)

[Supplementary Figure 1. Box Plots of Tau Peptide Concentrations by Alzheimer’s Disease Status. 9](#_Toc149758714)

[Supplementary Figure 2. Box plots of Tau Peptide Concentrations by CDR status. 9](#_Toc149758715)

[Supplementary Figure 3. Box plots of the fold-changes of the tau peptides by CDR status. 10](#_Toc149758716)

[Supplementary Figure 4. Box Plots of the plasma tau peptides concentrations by CERAD Neuritic Amyloid Plaque Score. 10](#_Toc149758717)

[Supplementary Figure 5. Box Plots of the plasma tau peptides concentrations by Braak Staging for NFTs. 11](#_Toc149758718)

[Supplementary Figure 6. Box Plots of the tau peptides concentrations in donors grouped as: controls (CERAD 0, Braak 0-II), PART (CERAD 0, Braak III-IV), AD (CERAD≥1, Braak III-IV) and Advanced AD (CERAD≥1, Braak V-VI). 11](#_Toc149758719)

[SUPPLEMENTARY REFERENCES 11](#_Toc149758720)

## SUPPLEMENTARY METHODS

## Immunoprecipitation Mass Spectrometry Quantification of Tau Peptides

One-ml plasma EDTA samples were thawed, vortexed for 30 seconds at 2000 rpm, and then centrifuged at 4,000 × g for 10 minutes. Automated IP was performed using the KingFisher Flex System (Thermo Fisher Scientific), incubating the samples with magnetic beads coupled with antibodies for 2 hours at room temperature. The samples were then washed with PBS, PBS 0.05% Triton X-100, PBS, 50 mM ammonium bicarbonate (AMBIC), and eluted with 0.5% formic acid. Additional tau enrichment was done by adding perchloric acid (PCA) (15 µl, 60% v/v) to the samples, which were briefly vortexed and incubated on ice for 15 minutes before centrifugation at 3,000 × g for 30 minutes at 4 °C. The supernatants were desalted using a 96-well SPE plate (Oasis PRiME HLB 96-well µElution Plate, 3 mg sorbent per well, Waters), lyophilized, and stored until trypsination. Trypsin (Sequencing grade, Promega) was dissolved and diluted to 2.5 µg/ml in 50 mM AMBIC, with 0.1 µg added per sample and incubated at 37 °C for 18 hours. Proteolysis was quenched with TFA final concentration of 0.1%. Samples were speed-vac-dry and stored at -20 °C until LC-MS analysis. The samples were resuspended in 50 µl of 0.01% TFA and analyzed in singlicates. Peptides were separated using liquid chromatography on an Ultimate 3000 nanoflow-LC (RSLC nano, Thermo Fisher Scientific), equipped with a trap column (300 μm i.d. × 5 mm packed with Acclaim PepMap 100 C18, 5 μm, Thermo Fisher Scientific) and a separation column (Easy Spray 75 μm i.d. × 500 mm, C18, 2 μm, 100 Å, Thermo Fisher Scientific). MS analysis was carried out using a hybrid Orbitrap mass spectrometer (Lumos, Thermo Fisher Scientific) equipped with a high-field-assymetric-waveform ion mobility separation (FAIMS) module and an EasySpray nano-ESI ion source. The optimized parameters for peptide detection and quantification have been described previously^1^. Endogenous peptide signals were normalized to heavy-isotope-labeled AQUA peptide standards, which were added to the samples during preparation. The light and heavy-labeled targeted peptides and the specific MS applied parameters are included in Supplementary Tables 1 and 2. The LC-MS data were analyzed using the software Skyline 22.1 (McCoss Lab, University of Washington). Samples from two brain donors were excluded from the analysis due to technical errors.

## SUPPLEMENTARY TABLES

## Supplementary Table 1. Tryptic tau peptides targeted in the study.

Respective dominant charge state, monoisotopic *m/z* value, optimal normalized collision energy (NCE) for higher energy collision-induced dissociation (HCD) and optimal compensation voltage (CV) used for FAIMS for each peptide are shown.

| **Phospho site** | **Peptide aa positions** | **Target peptide sequence** | **Charge state** | ***m/z*** | **NCE [%]** | **FAIMS CV (V)** |
| --- | --- | --- | --- | --- | --- | --- |
| T181 | 175-190 | TPPAPK[pT]PPSSGEPPK | 3 | 556.6062 | 25 | -70 |
| - | 195-209 | SGYSSPGSPGTPGSR | 2 | 697.321 | 25 | -50 |
| S199 | 195-209 | SGYS[pS]PGSPGTPGSR | 2 | 737.3039 | 25 | -50 |
| S202 | 195-209 | SGYSSPG[pS]PGTPGSR | 2 | 737.3039 | 25 | -50 |
| T205 | 195-209 | SGYSSPGSPG[pT]PGSR | 2 | 737.3039 | 25 | -50 |
| - | 212-221 | TPSLPTPPTR | 2 | 533.798 | 25 | -70 |
| T217 | 212-221 | TPSLP[pT]PPTR | 2 | 573.781 | 27 | -60 |
| T231 | 225-240 | KVAVVR[pT]PPKSPSSAK | 3 | 577.9887 | 30 | -60 |

## Supplementary Table 2. Heavy standards used for the nomalization of quantified peptides.

Respective monoisotopic m/z value, heavy labelling and spiked in concentrations are shown for each peptide. The heavy labeled amino acids are marked in bold. Dominant charge state, optimal normalized collision energy (NCE) for higher energy collision-induced dissociation (HCD) and optimal compensation voltage used for FAIMS are the same as for the corresponding light peptides shown in Supplementary Table 1.

| **Phospho site** | **Peptide aa positions** | **Target peptide sequence** | ***m/z*** | **Spiked in per sample (fmol)** |
| --- | --- | --- | --- | --- |
| T181 | 175-190 | TPPAPK[pT]PPSSGEPP**K** | 558.6129 | 1 |
| - | 195-209 | SGYSSPGSPGTPGS**R** | 702.325 | 0.5 |
| S199 | 195-209 | SG**Y**S[pS]PGSPGTPGSR | 742.3175 | 1 |
| S202 | 195-209 | SGYSSPG[pS]PGTPGS**R** | 742.3081 | 0.5 |
| T205 | 195-209 | SG**Y**SSPGSPG[pT]PGSR | 742.3175 | 0.1 |
| - | 212-221 | TPSLPTPPT**R** | 538.802 | 1 |
| T217 | 212-221 | TPSLP[pT]PPT**R** | 578.786 | 0.5 |
| T231 | 225-240 | **K**VAVVR[pT]PPKSPSSA**K** | 582.0022 | 1 |

|  | **Total Sample Set (N=123)** | **AD**  **(N = 69)** | **Non-AD**  **(N = 54)** | **P-value** |
| --- | --- | --- | --- | --- |
| **Neuropathology Characteristics** |  |  |  |  |
| Braak stage, n (%) | | | | |
| Stage 0 | 4 (3.3) | 0 | 4 (7.4) | <0.01 |
| Stage I/II | 25 (20.3) | 0 | 25 (46.3) |  |
| Stage III/IV | 34 (27.6) | 9 (13.0) | 25 (46.3) |  |
| Stage V/VI | 60 (48.8) | 60 (87.0) | 0 |  |
| CERAD neuritic plaque score, n (%) | | | | |
| None | 27 (22.0) | 0 | 27 (50.0) | <0.01 |
| Sparse | 29 (23.6) | 6 (8.7) | 23 (42.6) |  |
| Moderate | 28 (22.8) | 24 (34.8) | 4 (7.4) |  |
| Frequent | 39 (31.7) | 39 (56.5) | 0 |  |
| Thal Phase, n (%)  (Specific n for Thal staging only) | Total sample (n=67) | AD (n = 34) | Non-AD (n=33) |  |
| 0 | 10 (14.9) | 0 | 10 (30.3) | <0.001 |
| 1/2 | 9 (13.4) | 2 (5.9) | 7 (21.2) |  |
| 3 | 7 (10.4) | 1 (2.9) | 6 (18.2) |  |
| 4/5 | 41 (61.2) | 31 (91.2) | 10 (30.3) |  |
| Lewy body disease, n (%) |  |  | | |
| Brainstem predominant | 7 (5.9) | 4 (6.2) | 3 (5.6) | 0.16 |
| Limbic (transitional) | 8 (6.7) | 4 (6.2) | 4 (7.4) |  |
| Neocortical (diffuse) | 18 (15.1) | 11 (16.9) | 7 (13.0) |  |
| Amygdala predominant | 5 (4.2) | 4 (6.2) | 1 (1.9) |  |
| Olfactory bulb | 3 (2.5) | 4 (6.2) | 0 |  |
| Frontotemporal lobar degeneration, n (%) | 13 (10.6) | 6 (8.7) | 7 (13.0) | 0.56 |
| Chronic traumatic encephalopathy, n (%) | 3 (2.9) | 2 (3.7) | 1 (2.0) | 1.00 |
| Cerebral amyloid angiopathy, n (%) moderate-severe | 56 (45.5) | 42 (60.9) | 14 (25.9) | <0.01 |
| Arteriosclerosis, n (%) moderate-severe | 99 (80.5) | 56 (81.2) | 43 (79.6) | 0.83 |
| Atherosclerosis, n (%) moderate-severe | 47 (38.2) | 27 (39.1) | 20 (37.0) | 0.81 |
| Ratings of p-tau severity, n (%) moderate-severe |  |  |  |  |
| Dorsolateral frontal cortex | 59 (51.3) | 58 (90.6) | 1 (2.0) | <0.001 |
| Inferior orbitofrontal cortex |  |  |  |  |
| Superior temporal cortex | 66 (58.4) | 58 (93.5) | 8 (15.7) | <0.001 |
| Inferior parietal cortex | 55 (49.1) | 54 (88.5) | 1 (2.0) | <0.001 |
| CA1-hippocampus | 78 (70.9) | 57 (93.4) | 21 (42.9) | <0.001 |
| CA2-hippocampus | 61 (56.5) | 46 (75.4) | 15 (31.9) | <0.001 |
| CA4-hippocampus |  |  |  |  |
| Entorhinal cortex | 88 (77.9) | 60 (96.8) | 28 (54.9) | <0.001 |
| Amygdala | 72 (64.9) | 58 (95.1) | 14 (28.0) | <0.001 |

## Supplementary Table 3. Neuropathology Characteristics.

The 1997 NIA Reagan criteria were used for the neuropathological diagnosis of Alzheimer’s disease. Binary logistic regression compared donors with and without autopsy-confirmed Alzheimer’s disease on all outcomes. For semi-quantitative ratings of regional p-tau, cerebral amyloid angiopathy, arteriolosclerosis, and atherosclerosis, donors with moderate to severe ratings were grouped compared with donors who had no or mild severity ratings. Sample sizes: Due to missingness sample sizes are reduced for the following: dorsolateral frontal cortex, n = 115; superior temporal cortex, n = 113; inferior parietal cortex, n = 112; CA1-hippocampus, n = 110; CA2-hippocampus, n = 108; entorhinal cortex, n = 113; amygdala, n = 111. Abbreviations: AD = Alzheimer’s disease; CERAD = Consortium to Establish a Registry for Alzheimer’s Disease

## Supplementary Table 4. Associations between Plasma Tau Peptides and Ratings of p-Tau Severity at Autopsy

|  | **Frontal** | | **Superior Temporal** | | **Inferior**  **Parietal** | | **Entorhinal** | | **Amygdala** | | **Hippocampus** | |
| --- | --- | --- | --- | --- | --- | --- | --- | --- | --- | --- | --- | --- |
|  | **Est.** | **95% CI** | **Est.** | **95% CI** | **Est.** | **95% CI** | **Est.** | **95% CI** | **Est.** | **95% CI** | **Est.** | **95% CI** |
| p-tau181 | 0.35 | 0.10, 0.61 | 0.41 | 0.24, 0.59 | 0.38 | 0.20, 0.55 | 0.30 | 0.07, 0.43 | 0.32 | 0.13, 0.51 | 0.42 | 0.17, 0.68 |
| P-tau199 | 0.30 | 0.03, 0.56 | 0.30 | 0.11, 0.49 | 0.26 | 0.07, 0.44 | 0.11 | 0.0, 0.36 | 0.23 | 0.03, 0.43 | 0.16 | -0.12, 0.43 |
| P-tau202 | 0.17 | -0.11, 0.46 | 0.25 | 0.03, 0.47 | 0.21 | 0.00, 0.43 | 0.17 | -0.01, 0.35 | 0.17 | -0.01, 0.35 | 0.12 | -0.18, 0.41 |
| P-tau205 | 0.49 | 0.23, 0.75 | 0.42 | 0.25, 0.59 | 0.40 | 0.23, 0.57 | 0.35 | 0.07, 0.41 | 0.24 | 0.07, 0.41 | 0.46 | 0.19, 0.73 |
| P-tau205/tau195-209 | 0.44 | 0.24, 0.65 | 0.44 | 0.24, 0.65 | 0.49 | 0.33, 0.64 | 0.34 | 0.16, 0.49 | 0.44 | 0.27, 0.61 | 0.43 | 0.22, 0.65 |
| P-tau217 | 0.68 | 0.44, 0.92 | 0.61 | 0.44, 0.77 | 0.57 | 0.40, 0.73 | 0.54 | 0.15, 0.51 | 0.49 | 0.31, 0.67 | 0.66 | 0.41, 0.91 |
| P-tau217/tau212-221 | 0.77 | 0.58, 0.97 | 0.74 | 0.60, 0.88 | 0.68 | 0.53, 0.82 | 0.58 | 0.24, 0.59 | 0.59 | 0.41, 0.76 | 0.69 | 0.47, 0.92 |
| P-tau231 | 0.51 | 0.27, 0.76 | 0.49 | 0.33, 0.66 | 0.46 | 0.29, 0.62 | 0.45 | 0.09, 0.44 | 0.38 | 0.19, 0.56 | 0.57 | 0.32, 0.81 |
| Tau195-209 | 0.20 | -0.06, 0.46 | 0.28 | 0.10, 0.46 | 0.25 | 0.07, 0.43 | 0.19 | 0.07, 0.41 | 0.24 | 0.06, 0.43 | 0.21 | -0.05, 0.48 |
| Tau212-221 | 0.15 | -0.10, 0.40 | 0.23 | 0.05, 0.41 | 0.22 | 0.04, 0.40 | 0.18 | -0.03, 0.32 | 0.21 | 0.02, 0.40 | 0.18 | -0.08, 0.44 |

Multivariable linear regression examined the association between plasma tau levels and p-tau severity in the frontal cortex (dorsolateral frontal + inferior orbitofrontal), superior temporal cortex, inferior parietal cortex, entorhinal cortex, amygdala, and hippocampus (CA1+CA2+CA4). Models controlled for age at death, years between last blood draw and death, sex, and *APOE e4* carrier status. Abbreviations: Est. = standardized beta estimate; CI = confidence interval; p-tau = phosphorylated tau.

## SUPPLEMENTARY FIGURES


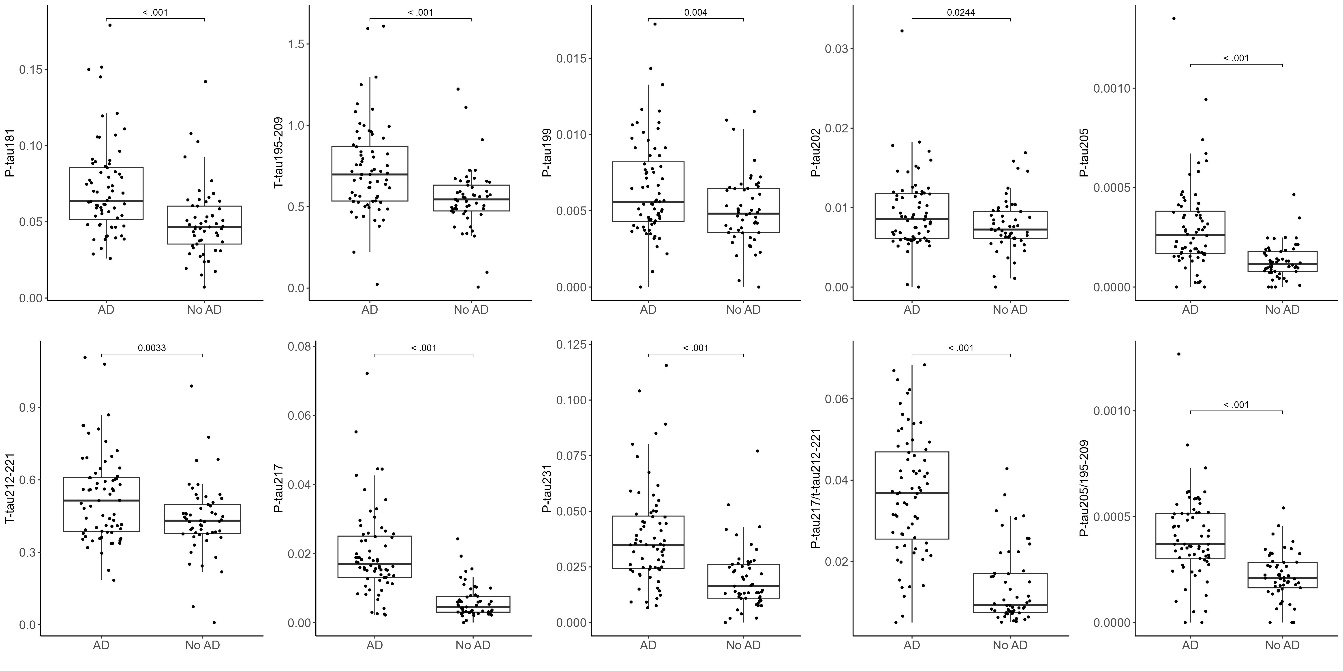


## Supplementary Figure 1. Box Plots of Tau Peptide Concentrations by Alzheimer’s Disease Status.

National Institute on Aging-Reagan Institute criteria were used for the neuropathological diagnosis of Alzheimer’s disease. Box plots include the median (bar) and interquartile range (whiskers) as well as the individual data points.


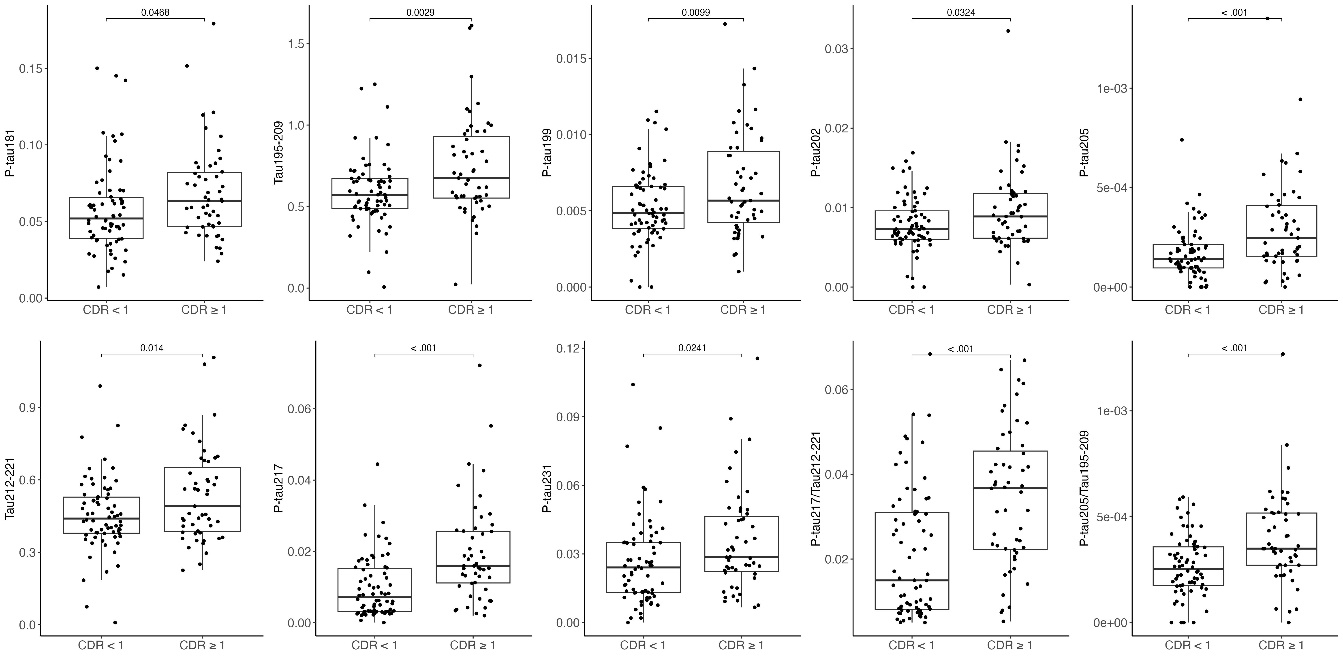


## Supplementary Figure 2. Box plots of Tau Peptide Concentrations by CDR status.

Box plots include the median (bar) and interquartile range (whiskers) as well as the individual data points. Red dots are brain donors with autopsy-confirmed Alzheimer’s disease.


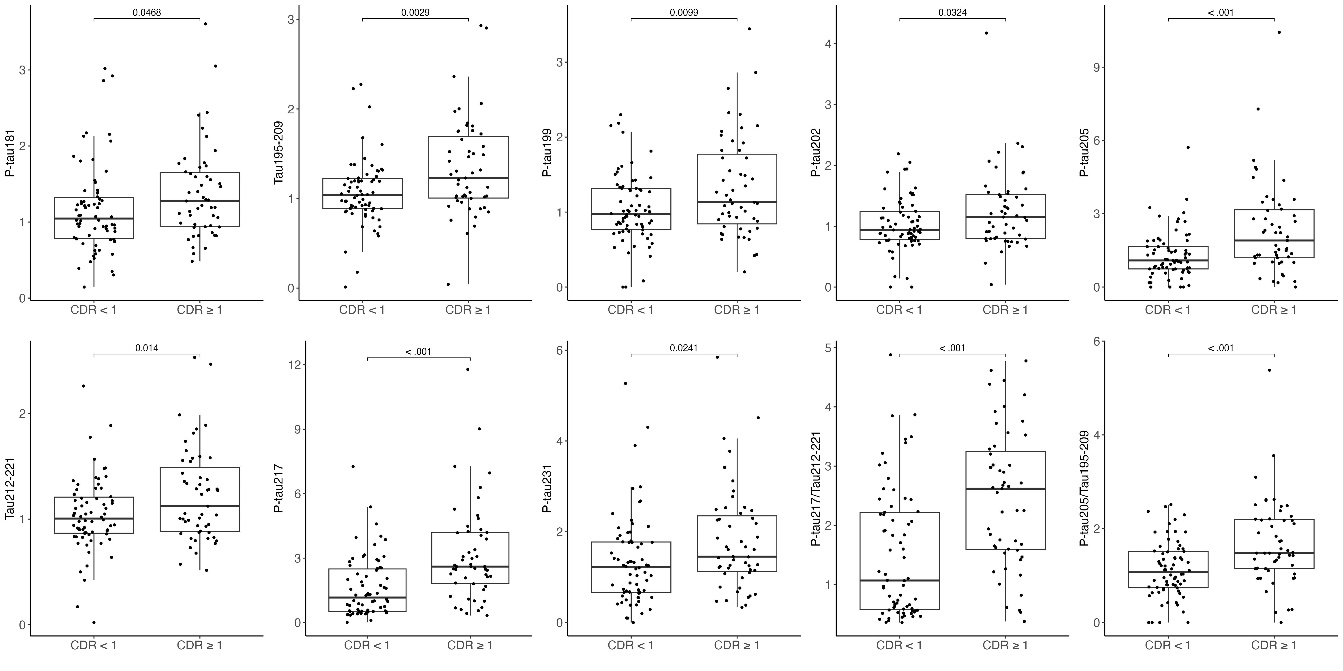


## Supplementary Figure 3. Box plots of the fold-changes of the tau peptides by CDR status.

The CDR<1 group was used as a reference. Box plots include the median (bar) and interquartile range (whiskers) as well as the individual data points. Red dots are brain donors with autopsy-confirmed Alzheimer’s disease.


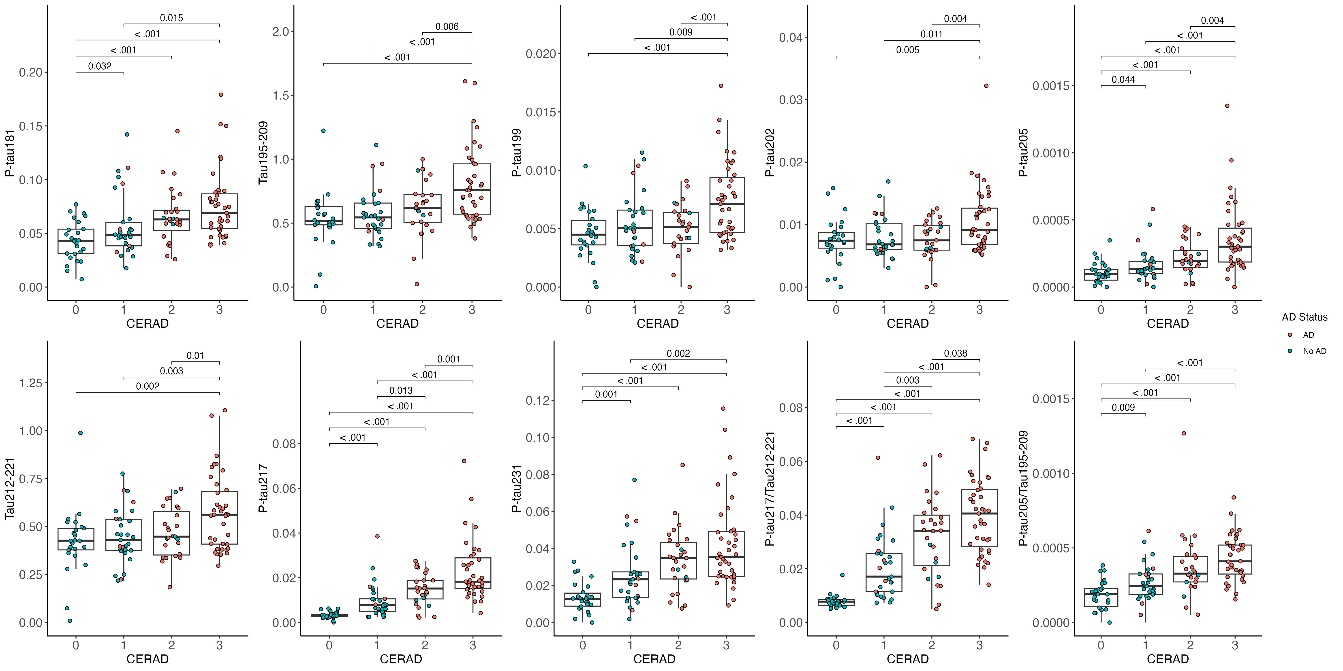


## Supplementary Figure 4. Box Plots of the plasma tau peptides concentrations by CERAD Neuritic Amyloid Plaque Score.

Box plots include the median (bar) and interquartile range (whiskers) as well as the individual data points**.** Participants are color-coded based on the presence (red) or absence (blue) of AD brain pathology.


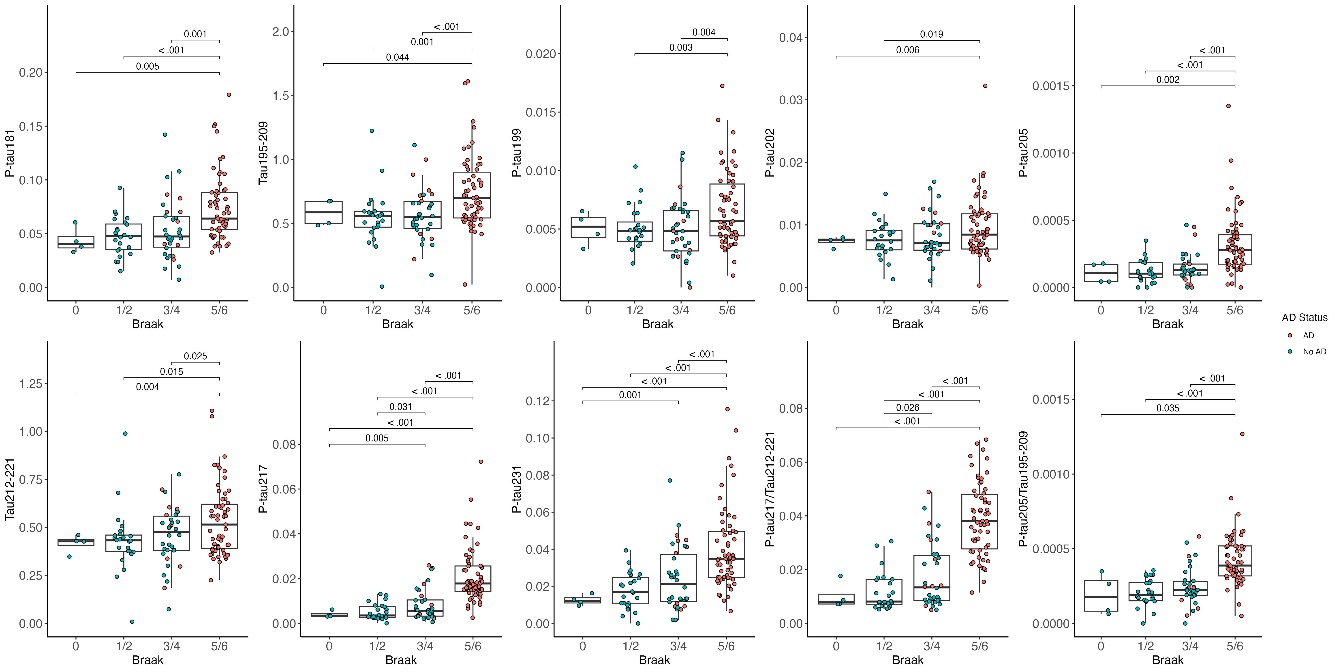


## Supplementary Figure 5. Box Plots of the plasma tau peptides concentrations by Braak Staging for NFTs.

Box plots include the median (bar) and interquartile range (whiskers) as well as the individual data points. Participants are color-coded based on the presence (red) or absence (blue) of AD brain pathology.


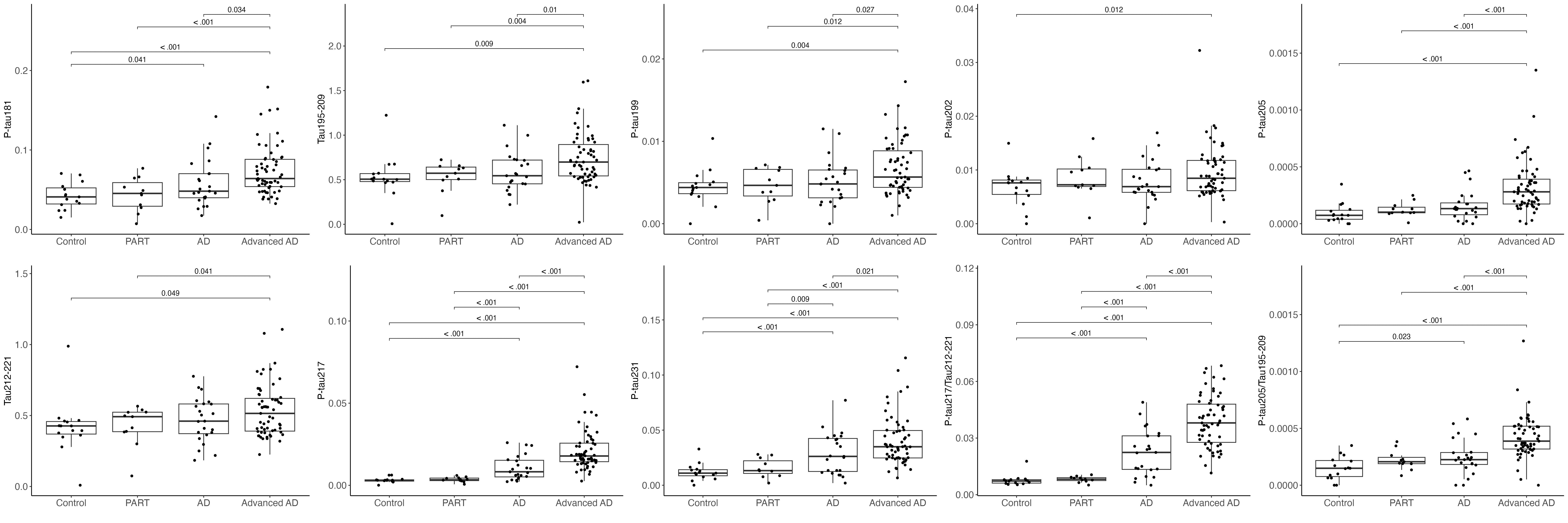


##

## Supplementary Figure 6. Box Plots of the tau peptides concentrations in donors grouped as: controls (CERAD 0, Braak 0-II), PART (CERAD 0, Braak III-IV), AD (CERAD≥1, Braak III-IV) and Advanced AD (CERAD≥1, Braak V-VI).

Box plots include the median (bar) and interquartile range (whiskers) as well as the individual data points.

## SUPPLEMENTARY REFERENCES

1. Montoliu-Gaya L, Benedet AL, Tissot C, et al. Mass spectrometric simultaneous quantification of tau species in plasma shows differential associations with amyloid and tau pathologies. *Nat Aging* 2023.
